# Development and content validity of the Barth Syndrome Symptom Assessment (BTHS-SA) for adolescents and adults

**DOI:** 10.1186/s13023-021-01897-z

**Published:** 2021-06-09

**Authors:** Chad Gwaltney, Jonathan Stokes, Anthony Aiudi, Iyar Mazar, Sarah Ollis, Emily Love, Alan Shields

**Affiliations:** 1 Gwaltney Consulting, 1 Bucks Trail, Westerly, RI USA; 2 Adelphi Values, 290 Congress St, 6th Floor, Boston, MA USA; 3 Stealth BioTherapeutics, 275 Grove Street, Suite 3-107, Newton, MA USA

**Keywords:** Content validity, Instrument development, Patient-reported outcomes, Barth Syndrome, Barth Syndrome Symptom Assessment

## Abstract

**Background:**

Barth Syndrome (BTHS) is a rare genetic disorder that presents as a complex of debilitating symptoms and reduced life expectancy. Well-developed, BTHS-specific assessments measuring primary signs and symptoms of BTHS are not currently available, making it difficult to evaluate treatment effects in BTHS clinical studies. The objective of this research was to develop symptom-focused patient-reported outcome (PRO) measures for use in clinical studies with adolescents and adults with BTHS.

**Methods:**

Concept elicitation interviews (CEIs) with pediatric (n = 18, age < 16 years) and adult (n = 15, age ≥ 16 years) individuals with BTHS and/or their caregivers were conducted to identify signs and symptoms relevant to BTHS and important to individuals with the condition. Based on CEI results, questionnaire construction activities were conducted to create unique adolescent and adult versions of the Barth Syndrome-Symptom Assessment (BTHS-SA). The questionnaires were evaluated in cognitive debriefing interviews (CDIs) with adolescents (n = 12; age 12- < 16 years) and adults (n = 12; age ≥ 16 years) with BTHS to assess relevance and readability of the tools.

**Results:**

During the CEIs, a total of 48 and 40 signs and symptoms were reported by the pediatric and adult groups, respectively; 31 were reported by both age groups. Fatigue/tiredness and muscle weakness were the symptoms most frequently reported by both pediatric and adult patients with BTHS as important to improve with an effective treatment. The CEI results informed construction of a nine-item version of the BTHS-SA for adolescents and an eight-item version for adults. Developed for daily administration, each version asks respondents to rate symptom severity “at its worst” over the 24 h prior to administration. CDIs with both adolescents and adults with BTHS demonstrated that each BTHS-SA version was reflective of the disease experience and that respondents could interpret the questionnaire as intended and provide responses that accurately reflected their symptom experience.

**Conclusions:**

The BTHS-SA adolescent and adult versions are content-valid PRO measures that can be used to evaluate severity of disease-specific symptoms in future clinical trials. Given the lack of available and well-developed assessments in this underserved therapeutic area, these tools fulfill a need for clinical researchers developing treatments for individuals with BTHS.

## Background

Barth Syndrome (BTHS) is a rare, X-linked genetic condition resulting from mutations in the TAZ gene that result in abnormal cardiolipin on the inner mitochondrial membrane. BTHS occurs almost exclusively in males [1–3]. While the prevalence of BTHS is not well documented [3], the disease is estimated to affect approximately 1 in every 300,000–400,000 individuals globally [1, 2] and, therefore, is considered to be an ultra-rare or orphan disease [4–6]. Patients with BTHS typically suffer from severe skeletal muscle myopathy and weakness, exercise intolerance, fatigue, hypotonia, early-onset cardiomyopathy (particularly left ventricular non-compaction), growth delay, and intermittent neutropenia [1–3]. Due to cardiomyopathy and/or infection associated with neutropenia, BTHS can be fatal early in life or result in a life expectancy of approximately 50 years for those who survive into adulthood [2, 3, 7]. The clinical features of BTHS often present in infancy, though the type and severity of signs and symptoms of the disease can be variable between individuals with the condition [1–3, 7]. Nevertheless, it is common for individuals with BTHS to develop skeletal myopathy, low muscle tone, and significant muscle weakness. In addition, extreme tiredness or fatigue is frequently experienced during periods of strenuous physical activity. At present, there are no treatments available for this life-threatening and life-limiting condition; rather, individuals with BTHS rely on supportive care and symptom management [8–10].

Research conducted in BTHS has thus far predominantly reported on clinical findings [11], rather than measuring symptom experiences from the patient perspective [12]. What is apparent, however, is that BTHS patients experience a wide range of symptoms and pathology. In 2018 the Barth Syndrome Foundation (BSF) convened to systematically gather patients’ perspectives on their condition and available therapies [13]. Despite the availability of the BSF patient report, there remains a paucity of literature documenting the disease-defining symptoms of BTHS from the perspective of individuals with the condition, and qualitative research is needed to better describe the BTHS experience. This information could provide important insights regarding (1) the experiences of BTHS from the perspective of individuals with the condition, (2) patient experiences that could be targeted in the development of novel treatments, (3) patient experiences that could be targeted in the development of new measures, and (4) endpoints that could be used in clinical trials of novel BTHS treatments.

A review of past clinical trials involving BTHS was conducted (using clincaltrials.gov), which found that rather than using a patient-reported outcome (PRO) measure, disease severity is often evaluated through clinical biomarkers and performance-based testing (e.g., exercise tolerance, functional performance, muscle strength, etc.) [14–16]. Although performance outcomes are useful measures of functioning, they do not provide a comprehensive measure of symptom experience from the patient’s perspective [14]. Non-specific tools (such as the Pediatric Quality of Life Inventory [PedsQL] [17], the Patient Reported Outcome Measurement Information System Fatigue Short Form [PROMIS F-SF] [18], and the Quality of Life in Neurological Disorders [Neuro-QoL] [19]) are available to assess symptom experience from the perspective of patients; however, no BTHS-specific PRO measures were identified that assess symptom severity. While non-specific PRO tools may measure some relevant BTHS experiences, they are not tailored to assess the most important and relevant signs and symptoms experienced by people with BTHS.

To create a more specific evaluation of the symptoms of BTHS and fill this need for patient-centric measures in BTHS, it is critical to obtain information directly from the target population on their symptom experience. With this information, a content-valid PRO measure could be developed for use in this underserved population. It is in this context that the Barth Syndrome – Symptom Assessment (BTHS-SA) was developed, with versions appropriate for use among adolescents and adults with the condition. The purpose of the present article is to describe the initial development of the BTHS-SA and evidence supporting its content validity as defined in regulatory guidance documents and expert guidelines (i.e., that the BTHS-SA targets relevant and important symptoms of BTHS and is both understood and used as intended by respondents [20–22]).

## Methods

This research was conducted in three stages: (1) concept elicitation, (2) concept selection and questionnaire construction, and (3) questionnaire content evaluation. Stage 1 involved qualitative concept elicitation interviews (CEIs) with 18 pediatric participants with BTHS (age < 16 years) and/or their caregivers and 15 adult participants with BTHS (age ≥ 16 years) and their caregivers if necessary. The purpose of these interviews was to identify and document the important and relevant sign and symptom concepts associated with the disease from the perspective of individuals with the condition and/or their caregivers. Findings from the CEIs were organized into a conceptual model depicting the proximal and increasingly distal concepts that characterize BTHS. In Stage 2, sign and symptom concepts were selected for measurement, and a PRO questionnaire was constructed to assess those concepts. Stage 3 involved the conduct of cognitive debriefing interviews (CDIs) with 12 adolescent participants with BTHS (age 12 to < 16 years) and 12 adult participants with BTHS (age ≥ 16 years) to evaluate the draft questionnaires for readability, comprehensibility, comprehensiveness, and relevance.

### Ethics and participant recruitment

Ethics review and approval for conducting the interviews was granted by the United States (US)-based Quorum Review Independent Review Board, and each study participant (and caregiver, if applicable) provided written informed consent prior to study enrollment. Participants for CEIs were recruited at the 2016 BSF Conference in Clearwater, Florida (US). Study participants for the CDIs were also identified and recruited through collaboration with the BSF between February and March 2017. For all age groups, individuals were considered ineligible for either stage of interviews if they had any condition that would interfere with their ability to participate in the interview or confound study results (e.g., prohibitive cognitive impairment, substance abuse).

For the Stage 1 CEIs and Stage 3 CDIs, the consenting and screening process for individuals older than 18 years of age and older consisted of the individual providing written or electronic informed consent followed by a self-reported diagnosis of BTHS via the study screening document. For both the CEIs and the CDIs, the caregivers of individuals younger than 18 years of age provided written or electronic informed consent and completed the screening document confirming the individual’s diagnosis of BTHS. Individuals between six and 18 years of age provided written or electronic assent documenting their willingness to participate in the interview. For the CEIs, in the pediatric cohort, caregivers were invited to participate in interviews depending on the individual’s age, ability to self-report, and level of comfort in the interview setting. With the exception of one adult and caregiver who requested to conduct the interview jointly, no caregivers of adults participated in these interviews. Due to the rarity of BTHS, individuals who participated in the CEIs were eligible to participate in the CDIs. Caregivers were not involved in the CDIs and all participants completed these interviews independently.

### Stage 1: Concept elicitation interviews

#### Conduct of interviews

All CEIs (N = 33) were conducted in two age-based populations consisting of pediatrics (n = 18; age < 16 years) and adults (n = 15; age ≥ 16 years). Each interview was conducted in person and in alignment with a semi-structured interview guide designed to give participants an opportunity to discuss their disease-related experiences freely and spontaneously. Additional prompts or targeted questions were utilized by interviewers to inform a deeper understanding of each sign or symptom, namely its severity, duration, and frequency. Supplementary interview activities were performed with participants to provide depth and clarity regarding the subjective importance and relevance of sign or symptom concepts reported by individuals with BTHS. For example, participants were asked to rate the degree to which each experienced sign or symptom concept bothered (i.e., felt annoyed or irritated by the sign/symptom), worried (i.e., felt troubled or concerned by sign/symptom), and impacted them (i.e., had a negative effect on daily activities/quality of life), as well as to rate the severity of each sign or symptom concept (i.e., how bad or serious) they experienced on a 0–10 numeric rating scale (where 0 = No bother/worry/impact or Not at all severe and 10 = Most bothersome/worrisome/impactful or Most severe). Participants were also asked to rank the top five most bothersome signs or symptoms of BTHS that would be important to improve with effective treatment (participants were permitted to name more than one most bothersome symptom if they reported multiple symptoms being equally the most bothersome in their experience).

#### Data management and analysis

All CEIs were audio-recorded, transcribed verbatim, and anonymized and coded using ATLAS.ti (ATLAS.ti Scientific Software Development GmbH, Berlin, Germany), a qualitative data analysis platform, to facilitate content analysis [23, 24]. Qualitative data coding was conducted to organize and catalog descriptions of participants’ experiences with signs or symptoms of BTHS as expressed during concept elicitation interviews. Coding followed an iterative process, whereby researchers read transcripts line-by-line, identifying transcript text and coding instances where participants reported a particular BTHS-related sign or symptom. A preliminary codebook was constructed following interview conduct and modified as the researchers coded the transcripts. In cases when an existing code from the codebook did not adequately characterize a segment of data, the research team came to consensus on the development of a novel code [23, 24]. Concept frequencies were determined by totaling the number of unique study participants that reported experiencing a specific sign or symptom. In instances where interviews were conducted with both individuals with BTHS and their caregivers (i.e., joint interviews), reports on the disease experience of the individual with BTHS were considered as one analytic unit (i.e., each dyad represented a single participant).

Saturation analyses were conducted to determine the adequacy of the study sample size and to determine whether further interviews would likely lead to the collection of new or important signs or symptoms associated with BTHS [20, 21, 25, 26]. These analyses were conducted separately for pediatrics and adults to evaluate saturation of concepts for each age-based cohort. Bother, worry, impact, and severity rating data were analyzed quantitatively to determine the mean rating and standard deviation (SD) per sign or symptom concept. Sign/symptom improvement ranking data were evaluated in two ways: (1) to determine the frequency of each symptom’s rank in the first through fifth position as most important to improve and (2) the total number of participants who ranked a sign or symptom in the top five most important to improve, regardless of position. Following analysis, the concepts elicited from participants were organized into a conceptual model depicting the BTHS disease experience.

### Stage 2: Concept selection and item generation

The construction of the BTHS-SA questionnaires, informed by best practices and regulatory guidance for questionnaire construction and use [20, 27], was initiated with the instrument development team in order to first reach consensus on the symptom concepts that would be targeted for measurement by the new PRO questionnaires for use in adolescent and adult BTHS populations. The concepts that emerged as most salient to pediatric (age < 16 years) and adult (age ≥ 16 years) participants were evaluated separately to inform concept selection in each target population. Of note, while the ages of pediatric patients whose experiences were captured in the CEIs ranged from 2.5 to 15 years of age, the resulting adolescent version of the BTHS-SA was designed to be used specifically by individuals 12–15 years of age. The development of the adolescent BTHS-SA for individuals 12–15 years of age was based on the need to provide an age-appropriate tool for adolescents in a clinical trial that enrolled individuals 12 years of age and older with BTHS. Once concepts were selected for inclusion, the development team created the format, specified the recall period, and structured the instructions, items, and response scales for each age-based questionnaire.

### Stage 3: Cognitive debriefing interviews

#### Conduct of interviews

CDIs (N = 24) were conducted either in person or over the telephone in two age-based populations, adolescents (n = 12; age 12–15 years) and adults (n = 12; age ≥ 16 years), using a semi-structured interview guide designed to assess individuals’ ability to understand and respond to the BTHS-SA (note: caregivers did not participate in CDIs to ensure that the adolescents with BTHS themselves could read, comprehend, and complete the BTHS-SA independently). To evaluate readability of the questionnaire, participants were asked to complete the BTHS-SA using a “think-aloud” process in which the participants read through instructions and item content out loud while articulating their interpretations of the questionnaire as they completed the assessment [28, 29]. This was followed by more structured questioning, in which participants were asked (1) to further elaborate on their interpretation of each component of the questionnaire (i.e., the instructions, items, and response options) based on the preliminary data provided during the “think-aloud” process, (2) to discuss the relevance of the concepts measured to their experience with BTHS, and (3) whether they would recommend any revisions to the questionnaires [20, 24]. Lastly, interviewers guided participants through an exercise to provide insight into the level of change that would constitute noticeable and/or important improvement on symptoms associated with BTHS. Each participant was asked to rate the smallest improvement in each item concept that would represent a (1) noticeable and (2) important change on the BTHS-SA response scales.

#### Data management and analysis

Following completion of data collection, audio recordings of the interviews were transcribed verbatim, anonymized, and coded using ATLAS.ti. Data were analyzed to identify any comprehension issues, difficult terms or phrases, or problems in selecting a response to any of the BTHS-SA items. The frequency of misinterpretations or suggested revisions to the questionnaire was documented by totaling the number of unique study participants who interpreted a component of the questionnaire in ways inconsistent with the questionnaire development team’s intentions or who recommended changes to questionnaire text. All participant reports regarding change score ratings were captured and entered into a dataset; means, SDs, and percentage change per item were calculated across study participants.

## Results

### Stage 1: Concept elicitation interviews

#### Concept elicitation interviews: participant demographic and health information

All CEIs were conducted in person in July 2016 and each interview lasted approximately 60 min. Of the 33 CEIs conducted with individuals with BTHS and/or caregivers, 18 were pediatrics (age < 16 years) and 15 were adults (age ≥ 16 years). A total of 21 interviews were conducted independently with individuals with BTHS (n = 7 pediatric, n = 14 adult), nine were conducted independently with caregivers (all of pediatric individuals), and three were conducted jointly with both individuals with BTHS and their caregiver (n = 2 pediatric [ages 12 and 15], n = 1 adult [age 26]). Pediatric participants’ ages ranged from 2.5 to 15.0 years (mean = 8.6 ± 3.9), with most being white (n = 15, 83.3%) and non-Hispanic (n = 18, 100.0%). The pediatric participants represented an international population that included individuals from the US, the United Kingdom (UK), France, Canada, and Italy. Of these study participants, eight (44.4%) had no concomitant health condition, though two (11.1%) each had depression/anxiety, high blood pressure, and/or migraine headaches (counts not mutually exclusive). In addition, six participants (33.3%) also reported having heart disease but, due to the nature of BTHS and its association with cardiomyopathy, this may be related to the BTHS itself and not a separate concomitant condition. An overview of demographic and self-reported symptom onset data can be seen in Table 1.

**Table 1:** Demographic and self-reported clinical characteristics of CEI and CDI participants

Participants	Mean age ± SD (range)	Race	Symptom onset (age)
Concept elicitation interviews (N = 33)			
Pediatrics (n = 18)	8.6 ± 3.9 (2.5–15.0)	White: n = 15 (83.3%)	< 6 yrs: n = 15 (83.3%)
		Black or African American: n = 1 (5.6%)	6–12 yrs: n = 3 (16.7%)
		Other: n = 2 (11.1%)	
Adults (n = 15)	22.9 ± 5.8 (16.0–34.0)	White: n = 14 (93.3%)	< 6 yrs: n = 13 (86.7%)
		Black or African American: n = 1 (6.7%)	≥ 18 yrs: n = 2 (13.3%)
Cognitive debriefing interviews (N = 24)			
Adolescents (n = 12) *Participants from the CEIs (n* = *3)*	13.8 ± 1.2 (11.97–15.45)	White: n = 9 (75.0%)	< 6 yrs: n = 12 (100.0%)
		Black or African American: n = 2 (16.7%)	
		Other: n = 1 (8.3%)	
Adults (n = 12) *Participants from the CEIs (n* = *8)*	22.9 ± 6.1 (16.0–34.9)	White: n = 12 (100.0%)	< 6 yrs: n = 10 (83.3%)
			6–12 yrs: n = 2 (16.7%)

Participant refers only to individuals with BTHS and not their interviewed caregivers

Among the 15 adult participants, ages ranged from 16.0 to 34.0 years (mean = 22.9 ± 5.8) and nearly all reported being white (n = 14, 93.3%) and non-Hispanic (n = 15, 100.0%). The adult participants also represented an international population inclusive of individuals from the US, UK, Canada, and Australia. Many adults (n = 6, 40.0%) reported no other health condition, though six (40.0%) reported depression/anxiety, three (20.0%) reported heart disease, and two (13.3%) reported high blood pressure.

### Concept elicitation interviews: signs and symptoms

**Fig. 1:**
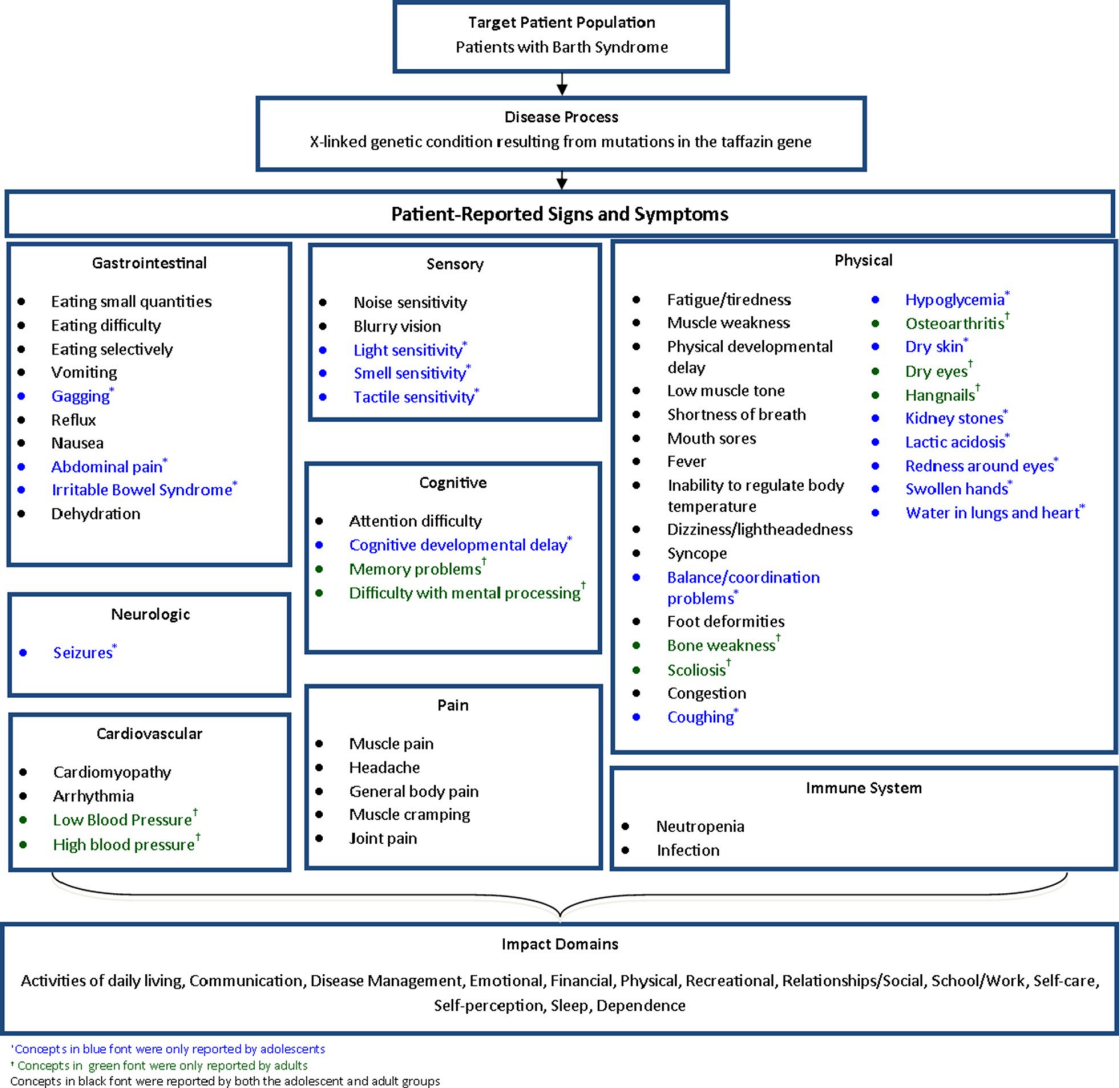
Barth Syndrome conceptual model

During the CEIs, a total of 48 and 40 signs and symptoms were reported by the pediatric and adult groups, respectively, totaling 57 unique signs and symptoms of BTHS identified across both age groups. Thirty-one (54.4%) of the 57 total concepts elicited from participants were reported by both age-based cohorts. The most frequently reported concepts across both cohorts were fatigue/tiredness (n = 32, 97.0%), cardiomyopathy (n = 27, 81.8%), muscle weakness (n = 26, 78.8%), and neutropenia (n = 19, 57.6%). Following analysis, these sign and symptom concepts were organized into a BTHS conceptual model (Fig. 1).

Additionally, during the CEIs, a total of 12 and 13 impact domains were reported by the pediatric and adult groups, respectively, resulting in a total of 12 unique impact domains reported by both pediatric and adult participants. Physical impacts (n = 32, 97.0%) were the most frequently reported impacts across the two ages groups, followed by school/work (n = 23, 70.0%), relationships/social (n = 22, 66.7%), emotional (n = 21, 63.6%), self-perception (n = 20, 60.6%), and activities of daily living (n = 17, 51.5%). The physical domain, which consisted of limitations to minimal daily activities (e.g., walking, lifting) as well as more strenuous physical activities (e.g., running, playing sports), was the domain that was most frequently reported by participants as being the most important to their experience with the impacts of BTHS (n = 10, 30.3). The only impact domain that did not overlap between pediatric and adult groups was the financial domain, which was only reported by adult participants (n = 2). The impact domains are included in the BTHS conceptual model (Fig. 1).

#### Pediatric population (n = 18)

Concept elicitation with pediatric participants and/or their caregivers yielded a total of 48 spontaneous expressions of sign and symptom concepts, all of which (100.0%) were reported in the first 75% of interviews, demonstrating saturation of concepts and confirming adequacy of study participant sample size. The most frequently reported concepts among the 18 pediatric participants included fatigue/tiredness (inclusive of exercise intolerance; reported by 17 pediatric participants, 94.4%), cardiomyopathy (n = 14, 77.8%), muscle weakness (n = 14, 77.8%), eating small quantities of food (n = 11, 61.1%), and physical developmental delay (n = 10, 55.6%). The most bothersome signs and symptoms were fatigue/tiredness (n = 9, 50.0%), headache (n = 4, 22.2%), eating difficulty (n = 3, 16.7%), and muscle weakness (n = 3, 16.7%). Muscle weakness (n = 10, 55.6%) and fatigue/tiredness (n = 8, 44.4%) were the signs and symptoms that participants most frequently reported as the most important to improve with an effective treatment. Headache (n = 4, 8.0 ± 0.82), muscle pain (n = 2, 7.5 ± 0.71), and fatigue/tiredness (n = 7, 6.86 ± 2.73) were reported with the highest mean severity ratings by participants. Of signs and symptoms rated by two or more participants, muscle cramping and fatigue/tiredness were reported as the highest mean bother rating, headache was reported as the highest mean worry rating, and headache, fatigue/tiredness, and muscle weakness were reported as the highest mean impact ratings. Note that, due to interview constraints (e.g., time limitations), not all participants who reported experiencing a sign or symptom concept provided a numeric rating to describe that concept’s bother, worry, and impact. Further details on the frequency with which pediatric participants reported individual signs and symptoms are provided in Table 2.

**Table 2:** Sign and symptom concept frequency: Pediatric reports (N = 18)

*Physical signs and symptoms*
Fatigue/tiredness (n = 17)*
Muscle weakness (n = 14)
Physical developmental delay (n = 10)
*Gastrointestinal signs and symptoms*
Eating small quantities (n = 11)
Eating selectively (n = 8)
Eating difficulty (n = 7)
*Immune system signs and symptoms*
Neutropenia (n = 7)
*Pain signs and symptoms*
Muscle pain(n = 5)
Headache (n = 5)

This table reflects symptoms reported by < 25% of participants (i.e., n ≥ 5)

^*^Total number of participants who reported sign or symptom

Pediatric participants (and, in some cases, their caregivers) reported a total of 12 impact domains, with the most frequently reported being the physical (n = 17, 94.4%), emotional (n = 12, 66.7%), and relationships/social (n = 11, 61.1%) domains, followed by the disease management, school/work, and self-perception domains (n = 10, 55.6% each). In addition to being the most frequently reported by participants, the physical domain was also the most frequently reported to be the most important to participants (n = 6, 33.3%). This domain consisted of impacts such as the inability to play or excel at sports, the inability to participate in physically active hobbies (such as bike riding, karate, or jumping on a trampoline), and the inability to keep up with siblings’ and friends’ walking pace. The emotional domain included feelings of sadness, frustration/anger, annoyance, worry, and intimidation, and the relationships/social domain consisted of difficulty visiting friends or family.

#### Adult population (n = 15)

Forty sign and symptom concepts were elicited spontaneously from the adult participants, 38 of which (95.0%) were elicited in the first 75% of interviews, demonstrating saturation of concepts and confirming adequacy of study participant sample size. The most frequently reported concepts among the 15 adult participants included fatigue/tiredness (inclusive of exercise intolerance; reported by 15 adult participants, 100.0%), cardiomyopathy (n = 13, 86.7%), muscle weakness (n = 12, 80.0%), neutropenia (n = 12, 80.0%), infection (n = 9, 60.0%), and arrythmia (n = 8, 53.3%). Further, fatigue/tiredness (n = 7, 46.7%), muscle weakness (n = 6, 40.0%), and neutropenia (n = 3, 20.0%) were reported as the most bothersome signs and symptoms, while fatigue/tiredness (n = 13, 86.7%) and muscle weakness (n = 9, 60.0%) were the signs and symptoms that participants most frequently ranked as most important to improve with an effective treatment. Of signs and symptoms rated by two or more participants, muscle pain and fatigue/tiredness were reported as the highest mean bother and worry ratings, and fatigue/tiredness and muscle pain were reported as the highest mean impact ratings. As with the pediatric bother, worry, and impact ratings, not all participants who reported experiencing a sign or symptom concept provided a numeric rating to describe that concept’s bother, worry, and impact. Further details on the frequency with which adult participants reported individual signs and symptoms are provided in Table 3.

**Table 3:** Sign and symptom concept frequency: Adult reports (N = 15)

*Physical signs and symptoms*
Fatigue/tiredness (n = 15)*
Muscle weakness (n = 12)
Shortness of breath (n = 5)
Mouth sores (n = 5)
Low muscle tone (n = 4)
*Immune system signs and symptoms*
Neutropenia (n = 12)
Infection (n = 9)
*Cardiovascular signs and symptoms*
Arrhythmia (n = 8)
*Pain signs and symptoms*
Muscle pain (n = 6)

This table reflects symptoms reported by < 25% of participants (i.e., n ≥ 4)

^*^Total number of participants who reported sign or symptom

Adult participants reported a total of 13 impact domains, with the most frequently reported being the physical (n = 15, 100.0%) domain, which was reported by all adult participants. This was followed by impacts in the school/work (n = 13, 86.7%), relationships/social (n = 11, 73.3%), and activities of daily living (n = 11, 73.3%) domains. The domains of self-perception (n = 10, 66.7%) as well as emotional impacts and dependence (n = 9, 60.0% each) were also frequently reported by adult participants. The physical domain was the most frequently reported to be the most important domain to adult participants (n = 4, 26.7%), and consisted of impacts such as difficulty being active, not having the energy to walk at a regular pace, and the inability to run. The school/work domain primarily consisted of challenges that individuals faced in working for pay, and the relationships/social domain included difficulty being socially active (e.g., going out with friends or dating). The main concepts reported in the emotional domain included feelings of sadness/depression and confusion due to lack of information regarding BTHS.

### Stage 2: Concept selection and questionnaire construction

Following concept elicitation, symptoms of BTHS were selected as targets for assessment in each age-based population, and two versions of the BTHS-SA, the adult and adolescent versions, were constructed as daily diaries for implementation in clinical trials on an electronic platform. While sign and symptom data were collected and aggregated from individuals with BTHS (and their caregivers) ranging in age from 2.5 to 15 years for the pediatric group, the BTHS-SA adolescent version was created to target individuals between ages 12 and 15 years of age (i.e., adolescents) due to decreased reliability of accurate self-report below age 12 [30]. The BTHS-SA adult version was developed for individuals with BTHS aged 16 years and older.

#### BTHS-SA adolescent version

Six symptom concepts were selected from the concept elicitation research for inclusion in the BTHS-SA adolescent version due to importance and relevance to adolescents with BTHS based on frequency of report and subjective level of bother, worry, impact, severity, and importance to improve with effective treatment. These concepts include tiredness, muscle weakness, muscle pain, early fullness when eating, difficulty eating, and headache, and they contributed to the creation of a nine-item adolescent version of the BTHS-SA questionnaire that asks respondents to evaluate symptom severity at its worst in a 24-h recall period using a five-point verbal response scale ranging from “No [concept] at all” to “Very severe [concept].” Three concepts (tiredness, muscle weakness, and muscle pain) were identified as needing to have their severity assessed during no activity (“At rest”) and activity (“During activity”) based on data collected from the CEIs wherein participants reported experiencing these symptoms of tiredness and muscle weakness both at rest (e.g., sitting or lying down) and during activities (e.g., exercising, walking, or climbing stairs), while muscle pain may be experienced at rest and due to activities (i.e., following the completion of activities). Though the sign and symptom concepts of cardiomyopathy, neutropenia, and physical developmental delay emerged as important and relevant concepts reported by adolescents, each was determined to be more accurately measured by clinical assessment and was not included in the BTHS-SA adolescent version.

#### BTHS-SA adult version

The following five symptom concepts were selected from the concept elicitation research as important and relevant to adults with BTHS based on frequency of report and subjective level of bother, worry, impact, severity, and importance to improve with effective treatment: tiredness, muscle weakness, muscle pain, dizziness/lightheadedness, and shortness of breath. These concepts served as the basis for the adult version of the BTHS-SA and are measured using an eight-item PRO questionnaire that instructs adults with BTHS to evaluate the severity of each symptom concept at its worst during the past 24 h on a five-point verbal rating scale ranging from “No [concept] at all” to “Very severe [concept].” Three concepts (tiredness, muscle weakness, and muscle pain) were identified as needing to have their severity assessed during no activity (“At rest”) and activity (“During activity”) based on data collected from the CEIs. While other sign and symptom concepts emerged as important and relevant (namely cardiomyopathy, arrhythmia, neutropenia, and frequent and long-lasting infections), similarly to the adolescent version of the BTHS-SA, these concepts were determined to be more accurately measured by clinical assessment than a PRO questionnaire and were not included in the BTHS-SA adult version.

### Stage 3: Cognitive debriefing interviews

#### Cognitive debriefing interviews: participant demographic and health information

A total of 24 participants (n = 12 adolescents [age 12–15 years]; n = 12 adults [age ≥ 16 years]) participated in individual 90-min CDIs between February and August 2017. This age range was selected for participation both to mirror the age range of a clinical trial population and to account for the variability in the accuracy of self-report below age 12. Eleven of these interviews (n = 3 adolescents and n = 8 adults) were conducted with individuals who had previously participated in the CEIs. All but one interview was conducted via telephone. Adolescent participants’ ages ranged 12–15 years, with a mean of 13.8 ± 1.2 years, and most reported being white (n = 9, 75.0%) and non-Hispanic (n = 11, 91.7%). Adult participants ranged from 16 to 34 years of age, with a mean of 22.9 ± 6.1 years, and all reported being white and non-Hispanic. All adolescent and adult CDIs included only individuals based the US. See Table 1 for demographic characteristics of the study participants.

### Cognitive debriefing interviews: questionnaire content evaluation results

#### Cognitive debriefing of the BTHS-SA (adolescent version)

Data elicited from CDIs support that adolescent participants were able to interpret the instructions, items, and response options of the BTHS-SA as intended. Participants were asked whether the questionnaire was easy or difficult to complete and whether they would recommend the addition of any other symptom concepts. All but one participant (n = 11, 91.7%) reported that the questionnaire was easy to complete, and seven participants (63.6%) reported that the BTHS-SA was comprehensive of their experience with BTHS. None of the additional symptoms recommended for addition to the BTHS-SA were reported by more than one participant. Additionally, of the questionnaire components (i.e., the instructions, items, or response options) that were not interpreted by CDI participants in accordance with the development team’s intentions and definitions, only a single participant in the CDIs demonstrated difficulty interpreting any one given component of the questionnaire. The only item-related interpretation issues were participants incorrectly defining the term “at rest” when used in items assessing “tiredness at rest” and “muscle pain at rest” (n = 1 each, 8.33%) where these participants interpreted “are rest” as relating to how tired one feels when going to bed. The majority of participants (n = 10, 83.3%) interpreted the 24-h recall period as intended; however, some participants (between one and three participants per item for Items 2–7 and 9) employed a different recall period in the context of providing responses to the items during cognitive debriefing (e.g., participants reported considering a longer timeframe than 24 h or did not use any timeframe when describing their response to an item). Table 4 presents interpretation results at an item level. In terms of concept relevance, all participants reported experiencing muscle weakness during activities either in the 24 h prior to completing the questionnaire or in their past experience with BTHS. At least half (n = 6, 50.0%) of the participants reported experiencing the remaining eight item concepts in the past 24 h or in their prior experiences with BTHS. Table 4 presents concept relevance results at an item level, noting the number of participants who reported experiencing the concept assessed by each item either within or outside the questionnaire’s recall period (24 h).

**Table 4:** Key indicators of content validity for the BTHS-SA, adolescent version

Item	Interpreted as intended (n, %)	Relevance*		Mean response selected, range^†^	Mean noticeable change rating^‡^ (SD)	Mean important change rating^§^ (SD)
		Experienced within the past 24 h	Experienced outside of the 24-h recall period			
Item 1: Tiredness at rest	9/11 (81.8%)	8/10 (80.0%);	1/10 (10.0%)	1.00, 0–2	1.00 (0.00)	1.00 (0.00)
Item 2: Tiredness during activities	11/12 (91.7%)	8/12 (66.7%);	3/12 (25.0%)	1.42, 0–2	1.25 (0.46)	1.56 (0.53)
Item 3: Muscle weakness at rest	10/11 (90.9%)	4/12 (33.3%)	5/12 (41.7%)	1.17, 0–4	1.0 (0.00)	1.14 (0.38)
Item 4: Muscle weakness during activities	8/11 (72.7%)	8/11 (72.7%)	3/11 (27.3%)	1.64, 1–3	1.14 (0.38)	1.50 (0.53)
Item 5: Muscle pain at rest	9/11 (81.8%)	3/11 (27.3%)	3/11 (27.3%)	0.82, 0–2	1.25 (0.50)	1.50 (0.58)
Item 6: Muscle pain due to activities	10/11 (90.9%)	7/12 (58.3%)	2/12 (16.7%)	1.25, 0–3	1.00 (0.00)	1.43 (0.53)
Item 7: Feeling of early fullness	9/12 (75.0%)	7/11 (63.6%)	1/11 (9.1%)	0.82, 0–2	1.00 (0.00)	1.40 (0.55)
Item 8: Difficulty eating	11/11 (100.0%)	1/12 (8.3%)	5/12 (41.7%)	0.67, 0–2	1.00 (0.00)	1.00 (0.00)
Item 9: Headache	9/12 (75.0%)	4/12 (33.3%)	6/12 (50.0%)	1.00, 0–2	1.00 (0.00)	1.50 (0.76)

^*^Number and percent of participants experiencing the symptom in the 24 h prior (and prior to the past 24 h) to assessment as reported during CDIs

^†^Severity as reported during CDIs on a five-point response scale (“No [CONCEPT] at all” to “Very severe [CONCEPT]”)

^‡^The minimum change on a five-point response scale that would represent a noticeable improvement in the symptom concept

^§^The minimum change on a five-point response scale that would represent an important improvement in the symptom concept

Participants most frequently reported that the muscle weakness during activities and at rest items represented the most important symptoms to evaluate for BTHS (n = 8, 80.0%; n = 5, 50%, respectively) and further rated the noticeable and important change required in each of the symptoms assessed by the BTHS-SA. As expected, all noticeable change ratings were smaller in magnitude than the important change ratings, with “tiredness during activities” (Item 2, n = 9, 1.56 ± 0.53), “muscle weakness during activities” (Item 4, n = 8, 1.50 ± 0.53), and “muscle pain at rest” (Item 5, n = 4, 1.50 ± 0.58) requiring the largest mean change on the five-point response scale to constitute an “important” change to the participants.

#### Cognitive debriefing of the BTHS-SA (adult version)

The data elicited from CDIs support that adult participants found the questionnaire easy to complete (n = 9, 75.0%) and comprehensive of their experience with BTHS (n = 7, 58.3%). Additionally, the data show that, overall, adult participants were able to interpret the instructions, recall period, items, and response options for the BTHS-SA as intended. In terms of additional symptoms suggested by participants for potential inclusion, there were minimal suggestions made (n = 5, 41.7%) with only one symptom (lack of appetite or getting full quickly, n = 2) being reported by more than one participant. Specifically, no more than a single participant in the CDIs demonstrated difficulty interpreting any given component of the questionnaire (i.e., the instructions, items, or response options) in accordance with the development team’s intentions and definitions. The only item-related interpretation issue was one participant interpreting the item concept of “muscle pain during activities” as “tiredness,” which was different than intended. All participants interpreted the 24-h recall period as intended; however, one participant (9.1%) reported using a longer recall period in the context of one of the items (“muscle weakness at rest”) during the cognitive debriefing. Table 5 presents interpretation results at an item level. All participants reported experiencing four of the item concepts assessed by the BTHS-SA (tiredness during activities, muscle weakness during activities, muscle pain due to activities, and shortness of breath) either in the 24 h prior to completing the questionnaire or in their past experience with BTHS. The majority of participants (at least n = 8, 72.7%) reported experiencing the remaining four concepts (tiredness at rest, muscle weakness at rest, muscle pain at rest, and dizziness/lightheadedness) in the past 24 h or in their prior experiences with BTHS. Table 5 presents concept relevance results at an item level, noting the number of participants who reported experiencing the concept assessed by each item either within or outside the questionnaire’s recall period (24 h).

**Table 5:** Key indicators of content validity for the BTHS-SA, adult version

Item	Interpreted as intended (n, %)	Relevance*		Mean response selected, range^†^	Mean noticeable change rating^‡^ (SD)	Mean important change rating^§^ (SD)
		Experienced within the past 24 h	Experienced outside of the 24-h recall period			
Item 1: Tiredness at rest	12/12 (100.0%)	10/12 (83.3%)	1/12 (8.3%)	2.00, 0–4	1.10 (0.32)	1.78 (0.83)
Item 2: Tiredness during activities	12/12 (100.0%)	n = 12/12 (100.0%)	0/0 (0.0%)	2.42, 1–3	1.36 (0.50)	2.10 (0.57)
Item 3: Muscle weakness at rest	10/11 (90.9%)	8/11 (72.7%)	0/0 (0.0%)	1.25, 0–4	1.14 (0.38)	1.50 (1.07)
Item 4: Muscle weakness during activities	12/12 (100.0%)	10/12 (83.3%)	2/12 (16.7%)	2.17, 0–4	1.18 (0.40)	1.82 (0.60)
Item 5: Muscle pain at rest	11/12 (91.7%)	6/11 (54.5%)	4/11 (63.4%)	1.64, 0–3	1.11 (0.33)	1.75 (0.71)
Item 6: Muscle pain due to activities	12/12 (100.0%)	9/12 (75.0%)	3/12 (25.0%)	2.08, 1–4	1.55 (0.69)	1.90 (0.74)
Item 7: Dizziness/‌lightheadedness	11/11 (100.0%)	8/12 (66.7%)	2/12 (16.7%)	1.42, 0–4	1.38 (0.74)	1.63 (1.06)
Item 8: Shortness of breath	12/12 (100.0%)	10/12 (83.3%)	2/12 (16.7%)	2.00, 1–3	1.25 (0.45)	1.82 (0.60)

^*^Number and percent of participants experiencing the symptom in the 24 h prior (and prior to the past 24 h) to assessment as reported during CDIs

^†^Severity as reported during CDIs on a five-point response scale (“No [CONCEPT] at all” to “Very severe [CONCEPT]”)

^‡^The minimum change on a five-point response scale that would represent a noticeable improvement in the symptom concept

^§^The minimum change on a five-point response scale that would represent an important improvement in the symptom concept

Participants most frequently reported that the items related to tiredness represented the most important symptom concepts to evaluate for BTHS (n = 10, 83.3% for tiredness during activities; n = 9, 75.0% for tiredness at rest) and further rated the noticeable and important change required in each of the symptoms assessed by the BTHS-SA. As expected, all noticeable change ratings were smaller in magnitude than the important change ratings, with “Tiredness during activities” (Item 2, n = 10, 2.10 ± 0.57) requiring the largest mean change on the five-point response scale to demonstrate an important change to the participants.

## Discussion

Individuals with BTHS experience a variety of debilitating signs and symptoms, including severe skeletal muscle myopathy and weakness, exercise intolerance, fatigue, hypotonia, early-onset cardiomyopathy (particularly left ventricular non-compaction), growth delay, and intermittent neutropenia [31]. There is an absence of available PRO measures to evaluate these experiences, and the BTHS-SA was designed to address this unmet need. While multiple existing instruments may measure some symptoms associated with BTHS, such as fatigue or pain (e.g., the PROMIS F-SF, PedsQL), none had been previously developed to capture the disease-specific symptoms of BTHS or tested in a BTHS population. The BTHS-SA was developed to assess the most relevant and important symptoms of BTHS from the perspective of adolescents and adults with the condition and was cognitively tested in these populations. After establishing the quantitative measurement characteristics of the BTHS-SA (e.g., reliability, convergent validity), it could be used to evaluate treatment benefit in clinical trials and may also be applied in observational research and real-world settings. In order to promote the applicability and suitability for use in regulated trials, the development of the BTHS-SA questionnaires adhered to measurement best practices and regulatory guidance; namely, the US Food and Drug Administration’s *Guidance for Industry – Patient-Reported Outcomes: Use in Medical Product Development to Support Labeling Claims* [20].

The qualitative research that provided the evidence to support the development of the questionnaire followed a rigorous methodology. Findings from qualitative concept elicitation research suggest that, while individuals with BTHS experience numerous and variable signs and symptoms, there is a defined set of salient concepts that best characterize the disease for pediatrics and adults. The identification of these symptoms from the perspective of individuals with the disease informed the development of the BTHS-SA. CDIs demonstrated that the questionnaires are relevant and understandable to individuals with the condition, supporting the content validity of the tool.

Several key symptoms emerged as important to the experience of BTHS from the patient perspective, which informed the selection of measurement concepts for the BTHS-SA. The relevance of fatigue, tiredness, and muscle weakness in the pediatric and adult populations was clear. In both the pediatric and adult age groups, the symptom of fatigue/tiredness was the most frequently reported concept. It was also reported to be the most important symptom to improve with an effective treatment by both age groups and was rated highly relative to other concepts in terms of its bother, worry, and impact for individuals with BTHS. Also emerging as a key concept for both the pediatric and adult populations was muscle weakness, which was frequently reported and rated as one of the most important signs or symptoms to improve.

BTHS was most impactful to individuals in terms of posing limitations to physical functioning, which ranged from difficulty walking and lifting objects to a reduced ability to participate in physical activities such as sports. Nearly all pediatric and adult participants reported experiencing physical impacts associated with BTHS, and individuals stated that these impacts were the most important to their experience with the condition. These physical impacts were predominantly triggered by experiences of fatigue/tiredness and/or muscle weakness, further corroborating these symptoms as being central to BTHS, as they impacted individuals in their daily lives.

Findings from the CDIs support the readability, comprehensibility, comprehensiveness, and ease of completion for both the BTH-SA adult and adolescent versions and demonstrate that that the content of each is comprehensive of the experience of the target age group of individuals with BTHS. Subsequently, no revisions were made to either version of the questionnaire. More specifically, during the CDIs all adolescent participants reported experiencing muscle weakness during activities and nearly all reported experiencing tiredness (both at rest and during activities). All adult participants also reported experiencing tiredness and muscle weakness, as well as muscle pain and shortness of breath, either within or prior to the 24-h recall period of the questionnaire. The items for muscle weakness and tiredness were reported by both age groups as being the most important to measure in relation to BTHS, and the items relating to tiredness and muscle weakness during activities were reported to require the largest average decreases on the verbal response scale to constitute an important improvement (2.10 and 1.56 [adult and adolescent] and 1.82 and 1.50 [adult and adolescent], respectively). It should be noted that the mean severity ratings of tiredness and muscle weakness (2.42 and 2.17, respectively) were the highest of all the items (the mean severity rating of the remaining items ranging from 1.25 to 2.08), allowing for the greatest improvement in severity on the response scale, which was frequently described by participants as progression to “No [concept] at all.”

Thus, among the numerous symptoms and impacts that individuals with BTHS reported during the interviews, the evidence from both stages of research supports fatigue/tiredness and muscle weakness as being the cardinal symptoms of the condition from the patient perspective, in terms of relevance and importance across age groups, as well as impact. Disease-specific tools tailored to the experience of BTHS are warranted, given the unique combination of symptoms that this patient group may experience. In particular, fatigue/tiredness and muscle weakness may serve as more specific targets for measuring treatment benefit from the patient perspective, given their importance in the BTHS experience.

### Limitations

The first stage of research (i.e., concept elicitation) afforded a global perspective of the BTHS experience, as participants from various countries participated in the interviews; however, the third stage of research (i.e., cognitive debriefing) included only individuals living in the US. As BTHS is a disease that affects individuals globally and the BTHS-SA may be used to support research conducted outside of the US, it may be useful to conduct debriefing interviews with individuals with BTHS living in other countries. This could be achieved as part of the linguistic validation and translation process, rather than as multiple stand-alone interview studies [32]. Future research in concordance with good practices in translation and cultural adaption is also warranted [32]. As the BTHS-SA was developed using input of various nationalities, there is evidence to support the tool’s content validity in a global population; however, further refinement may be needed for confirmation. The present research on the BTHS-SA supports its content validity, a critical characteristic for measurement. Evidence regarding other measurement characteristics (e.g., reliability, convergent/discriminant validity) of the BTHS-SA and potential item deletion or modification – using analyses that are appropriate for ultra-rare conditions and small sample sizes, such as descriptive statistics and bivariate correlations – will be made available in a subsequent publication. Additionally, the qualitative data should be augmented with quantitative data regarding the threshold for meaningful within-patient change or responder definition. Furthermore, while the content validity in pediatrics < 12 years of age is supported (as the CEIs included participants 2.5 years and older and/or caregivers), if the assessment tool is utilized in populations younger than 12 years of age, modifications to the BTHS-SA would need to be considered [33], as there is currently no data available to support the readability, comprehensibility, comprehensiveness, and ease of completion of the adolescent BTHS-SA among pediatric subjects < 12 years of age [33].

## Conclusions

In conclusion, results from the qualitative research described herein, along with the results of the quantitative performance characteristic evaluation (to be provided in a separate publication), support the BTHS-SA as a content-valid and psychometrically sound BTHS symptom-focused PRO questionnaire for use in clinical trials of investigational treatments.

## Data Availability

Data generated or analyzed during this study are either included in this published article or are available from the corresponding author upon reasonable request.

## Abbreviations

BSF: Barth Syndrome Foundation
BTHS: Barth Syndrome
BTHS-SA: Barth Syndrome-Symptom Assessment
CDI: Cognitive debriefing interview
CEI: Concept elicitation interview
Neuro-QoL: Quality of life in neurological disorders
PedsQL: Pediatric Quality of Life Inventory
PRO: Patient-reported outcome
PROMIS F-SF: Patient Reported Outcome Measurement Information System Fatigue Short Form
SD: Standard deviation
UK: United Kingdom
US: United States
